# *Amelanchier* Medik. Species: An Underutilized Source of Bioactive Compounds with Potential for Pharmacological and Nutraceutical Applications

**DOI:** 10.3390/molecules30173562

**Published:** 2025-08-30

**Authors:** Sandra Saunoriūtė, Justinas Sukackas, Lina Raudonė

**Affiliations:** 1 Department of Pharmacognosy, Faculty of Pharmacy, Lithuanian University of Health Sciences, Sukileliu Av. 13, LT-50162 Kaunas, Lithuania; sandra.saunoriute@lsmu.lt (S.S.); justinas.sukackas@lsmu.lt (J.S.); 2 Research Institute of Natural and Technological Sciences, Vytautas Magnus University, LT-40444 Kaunas, Lithuania; 3 Laboratory of Biopharmaceutical Research, Institute of Pharmaceutical Technologies, Lithuanian University of Health Sciences, Sukileliu Av. 13, LT-50162 Kaunas, Lithuania

**Keywords:** *Amelanchier*, bioactive compounds, traditional uses, nutraceutical, pharmacology

## Abstract

*Amelanchier* Medik. (*Rosaceae*) is a genus of perennial, deciduous shrubs and trees distributed across temperate and boreal regions of the Northern Hemisphere. Traditionally, Native American communities used fruits, leaves, bark, and roots to treat digestive ailments, fevers, colds, inflammation, and to promote general well-being. Scientific investigation began with molecular studies in 1946 and phytochemical research in 1978, with research activity on some *Amelanchier* species increasing substantially between 2010 and 2025. Fruits are rich in phenolic compounds—particularly flavonoids and anthocyanins—along with triterpenes, carotenoids, vitamins, and minerals. Pharmacological studies of selected species report antioxidant, anti-inflammatory, anticancer, antidiabetic, antibacterial, and antiviral activities. Despite extensive chemical profiling in several members of the genus, the biological and toxicological properties of *Amelanchier* remain insufficiently explored, and clinical evidence is lacking. This review synthesizes recent findings on the phytochemistry, medicinal applications, and biological effects of *Amelanchier* species, linking traditional knowledge with modern research and highlighting priorities for future biomedical investigation.

## 1. Introduction

*Amelanchier* Medik. genus belongs to *Rosaceae* Juss. family and, according to The World Online (WFO) database, it currently comprises 27 known accepted species: *A. alnifolia* (Nutt.) Nutt. ex M. Roem., *A. amabilis* Wiegand, *A. asiatica* (Siebold & Zucc.) Endl. ex Walp., *A. arborea* (F. Michx.) Fernald, *A. bartramiana* (Tausch) M. Roem., *A. canadensis* (L.) Medik., *A. cretica* (Willd.) DC., *A. cusickii* Fernald, *A. fernaldii* Wiegand, *A. gaspensis* (Wiegand) Fernald & Weatherby, *A. humilis* Wiegand, *A. interior* E.L.Nielsen, *A. intermedia* Spach, *A. × lamarckii* F. G. Schroed., *A. laevis* Wiegand, *A. nantucketensis* E. P. Bicknell, *A. × neglecta* Eggl. ex K. R. Cushman, M. B. Burgess, E. T. Doucette & C. S. Campb., *A. ovalis* Medik., *A. pallida* Greene, *A. parviflora* Boiss., *A. sanguinea* (Pursch) DC., *A. sinica* (C. K. Schneid.) Chun., *A. × spicata* (Lam.) K. Koch, *A. stolonifera* Wiegand, *A. turkestanica* Litv., *A. utahensis* Koehne, *A. × quinti-martii* Louis-Marie [1].

Plants of the *Amelanchier* Medik. genus are commonly referred to as “shadbush” and are also known as “saskatoon” or “serviceberry”. These perennial shrubs and trees originate from Canada and the northern regions of the United States. It has been naturalized in various areas, including Africa, Europe, and the eastern part of Asia [2,3,4]. *Amelanchier* species are highly adaptable and can thrive in different types of soil and environmental conditions [5]. This trait, while beneficial for cultivation, may allow them to outcompete native plants in certain regions. Due to the high level of genetic variability within the genus and the overlapping ranges of many species, natural hybridization in the *Amelanchier* genus is relatively common [6]. *A. alnifolia* (Nutt.) Nutt. ex M. Roem., commonly known as ʺSaskatoon berryʺ, is the main representative species of the genus. Along with *A. canadensis* (L.) Medik. (Canadian serviceberry), *A. arborea* (F. Michx.) Fernald has attracted significant attention from both the pharmaceutical and food industries [3,7,8,9]. Native Americans use different parts of *Amelanchier* in traditional medicine. The fruits, bark, and roots have been used for various medicinal purposes, including treating digestive issues, colds, and inflammation [3]. Early studies (pre-2000) focused on botanical taxonomy and ethnobotanical uses, while recent research (post-2010) has shifted towards phytochemical characterization and bioactivity testing. *Amelanchier* species have gained attention due to their rich content of phenolic compounds, flavonoids, and triterpenoids, which have demonstrated promising pharmacological effects. In recent years, the antioxidant, anti-inflammatory, antibacterial, anti-allergic, anti-diabetic, anticancer, anti-atherosclerotic, antitumor, and neuroprotective effects of *Amelanchier* have been proven [10,11,12,13,14,15]. Their chemical diversity suggests that potential applications in functional foods and phytotherapy are still underutilized.

Indeed, *Amelanchier* represents a unique combination of ornamental, ecological, nutritional, and phototherapeutic potential that remains underrepresented in scientific literature compared with other fruit-bearing genera. At the same time, certain species such as *A. × spicata* have demonstrated invasive potential outside their native range, highlighting the need for careful ecological management alongside potential biomass valorization. Plants remain the primary origin of many phenolic, triterpenoid, and carotenoid compounds of interest in functional food and phytotherapy research, and *Amelanchier* combines this phytochemical richness with broad ecological adaptability and cultural significance. While the genus has a long history of traditional use and is increasingly cultivated for its edible fruits, research on its chemistry, biological effects, and safety is scattered or disproportionately concentrated on a few species. This review aims to provide a comprehensive examination of the current research on the phytochemical properties and biological effects of the *Amelanchier* Medik. genus. It highlights key findings, identifies scientific uncertainties, and explores emerging trends and gaps to offer valuable directions for future studies.

## 2. Results and Discussion

### 2.1. Nomenclature and Taxonomy

The genus *Amelanchier* Medik. was first described in 1789 by the German botanist Friedrich Casimir Medicus [16]. Its taxonomy is notably complex, primarily due to frequent hybridization, polyploidy, and apomixis [17]. Hybridization occurs naturally and in cultivation, leading to numerous intermediate forms that complicate species identification and classification [6]. Initially, the genus *Amelanchier* was documented with 18 species in a monograph by Jones (1946) and in modern floristic reviews by Kartesz and Kartesz (1980) [18,19]. Krüssmann (1976) later reported 23 *Amelanchier* species in North America. Additionally, one European species (*A. ovalis* Medik.) and one Asiatic species (*A. asiatica*) have been identified [20,21]. While most species are concentrated in North America, a few, such as *A. sinica*, are endemic to China. Other species, like *A. asiatica*, are found in East Asia, including China, Korea, and Japan [22]. Currently, World Flora Online recognizes 27 species within the genus *Amelanchier*, including four natural hybrids [1].

According to historical records, the first *Amelanchier* species, *A. canadensis*, was introduced to Europe in the 17th century [23]. Another *Amelanchier* species, *A. × spicata*, whose origins remain a topic of debate, emerged in Europe during the 18th and 19th centuries [17]. Since Fernald (1946) demonstrated that *A. × spicata* is not part of the American flora, it likely originated in Europe as a derivative of *A. alnifolia* and *A. humilis* [24,25]. This hypothesis suggests that the species may have developed through cultivation or hybridization after being introduced to Europe [26]. Throughout the 20th century, multiple reports documented the naturalization of *A. × spicata* in various plant communities across Western Europe, indicating its successful adaptation to new environments. These accounts highlight *A. × spicata’s* remarkable ability to integrate into local ecosystems, gradually expanding beyond cultivated areas and successfully establishing self-sustaining populations in the wild [25]. Its adaptability to diverse environmental conditions suggests that the species has not only survived but also thrived in its introduced range, potentially competing with native vegetation. According to Schroeder (1970), two other *Amelanchier* species, *A. × lamarckii* and *A. canadensis*, also became naturalized in Europe. Their successful establishment further demonstrates the genus *Amelanchier*’s ability to adapt to European landscapes. The presence of these species in natural ecosystems suggests they may contribute to local biodiversity while also raising questions about their ecological impact, interactions with native flora, and potential for further range expansion. If *A. canadensis* is the ancestor of *A. × lamarckii*, which has been known in Europe since 1850, then *A. canadensis*, recorded in Sweden as early as 1830, likely originated from *A. alnifolia*, possibly with genetic influence from *A. arborea*. In Great Britain, the naturalized species *A. × spicata*, *A. × lamarckii*, and *A. canadensis* predominantly grow on acidic soils [27]. These three *Amelanchier* species have become the most successfully adapted to the environmental conditions of their secondary habitat in Europe. By the middle of the 20th century, *A. × spicata* had spread extensively across Lithuania, Latvia, Estonia, Poland, Russia, and reached as far as the Far East [17].

### 2.2. Phytochemical Composition of Amelanchier Species

*Amelanchier* species are rich in various bioactive compounds, such as flavonoids and phenolic acids. The most abundant flavonoids found in these species are proanthocyanidins, anthocyanins, and flavonols [7,15,28,29,30,31]. Phenolic acids, including caffeic acid, chlorogenic acid, coumaric acid, and ferulic acid, are abundant in plant materials [7,30,31,32,33]. Furthermore, the plant materials were found to be rich in various vitamins, minerals, and sugars, including glucose, fructose, and sorbitol [34,35]. Organic acids, such as citric, malic, oxalic, quinic, succinic, and tartaric acids, were detected in tested *Amelanchier* fruit samples [7,31,36,37]. The sugar-to-acid ratio determines fruit taste and consumer acceptability [38]. Most *Amelanchier* fruits present a mild and sweet flavor profile [39]. Mikulic-Petkovsek et al. determined that *A. canadensis* fruits have the highest sugar-to-acid ratio of 14.39, therefore having the highest sweetness perception compared to other commonly consumed fruits [40]. This characteristic is important in the development of new categories of functional foods [41]. Triterpenoids such as betulinic, oleanolic, and ursolic acids were detected in the plant material, with oleanolic acid being the most abundant triterpenoid in the fruit [38]. The major carotenoid compounds were β-carotene and lutein, and there were lower amounts of zeaxanthin [38,42,43]. Non-toxic levels of cyanogenic glycosides, including amygdalin and prunasin, were also detected [43]. The phytochemical diversity of certain *Amelanchier* species is well-documented, particularly their high content of phenolic compounds, flavonoids, triterpenoids, and carotenoids. However, there remains significant variation in the reported compound profiles, likely due to differences in extraction methods, geographical origin, and plant maturity at the time of collection.

In the context of a changing climate and environmental conditions, both the phytochemical profile and adaptability of species also change. Furthermore, many invasive species are stress-resistant, and their adaptation strategies may lead to a higher concentration of protective phytochemicals (e.g., antioxidants, stress-related polyphenols). Understanding how environmental factors influence bioactive compound production could enhance their targeted application in medicine and nutrition. Phytochemical analyses demonstrate that *Amelanchier* species contain a rich profile of phenolic compounds, usually extracted from their fruits. The phenolic content varies among species endemic to specific regions. Most research has focused on *A. alnifolia*, *A. canadensis*, and *A*. × *lamarckii*, while many other species remain largely unstudied. Some endemic species (*A. nantucketensis*, *A. bartramiana*, *A. interior*, *A. humilis*, *A. sanguinea*, *A. ovalis*) may contain unique phenolic compositions that are worth investigating. As particular species are considered invasive in many countries, further research could explore potential new medicinal applications for the genus. On the other hand, many *Amelanchier* species remain uninvestigated, and their phytochemical profiles are still unexplored. Further studies could focus not only on the known phytochemicals but also towards novel or minor constituents as they possibly carry chemophenetic significance. Environmental factors, diverse habitats, and adaptability shape the phytochemical profiles, but these factors are subject to scientific uncertainties.

#### 2.2.1. Hydroxycinnamic Acid Content of *Amelanchier* Species

The accumulation of these compounds varies between the species and different parts of the same plant [34,35]. *A. alnifolia* fruits were found to be one of the highest in total phenolic acid content (59 mg/100 g) out of 24 fruits tested, along with chokeberry, blueberry, and sweet rowanberry (96.85 and 75 mg/100 g) [44]. In the fruits of *A. × lamarckii*, 45 different phenolic compounds were determined. Most studies agree that hydroxycinnamic acids, especially chlorogenic acid, are the predominant phenolic acids in *Amelanchier* fruits. Hydroxycinnamic acids were found to be the predominant phenolic compounds, which represented 47% of the total phenolics analyzed. Chlorogenic acid represented 93% of all hydroxycinnamic acids [31,32,33]. It was more abundant in the fruits rather than the plant leaves (190.64 mg/g vs. 18.16 mg/g) [8]. Coumaric, ferulic, and caffeic acids were also identified in the plant materials. *A. alnifolia* fruits were found to contain higher levels of hydroxycinnamates (70–178 mg/100 g of fruit weight) than common fruits like apple (5.3 mg/100 g of fruit weight), plum (6.8 mg/100 g of fruit weight), nectarine (6.8 mg/100 g of fruit weight) and peach (4.8 mg/100 g of fruit weight) [9,45]. Scientific uncertainties remain within the alliterations in the profiles depending on seasonal and ontogenetic factors across all the possible plant materials.

#### 2.2.2. Flavonoid and Proanthocyanidin Content of *Amelanchier* Species

The primary anthocyanin identified was cyanidin-3-galactoside, accounting for 90% of the total anthocyanin content [9,31,46]. The other anthocyanins identified were cyanidin-3-glucoside, cyanidin-3-xyloside, and peonidin-3-glucoside, though they were present in lower amounts, at 80 mg/kg of fruit. The individual anthocyanin profiles in fruits of the *Amelanchier* genus vary and have chemophenetic significance. In *A. alnifolia* fruits, peonidin-3-glucoside was not detected. The ripe fruit exocarp and the seed coating exhibited the highest accumulation of anthocyanins, proanthocyanins, and flavonols [9,33,46]. A correlation was found between fruit pH and anthocyanin content, indicating that more acidic fruits tend to have higher anthocyanin levels and appear darker in color [7,29]. *A. alnifolia* was found to have greater amounts of total anthocyanins (562.4 mg/100 g) than raspberries, chokecherries and strawberries (365.2 mg/100 g, 177.39 mg/100 g, and 97.5 mg/100 g) [47].

The main flavonols identified in the fruit include quercetin diglycosides (quercetin 3-O-rutinoside, quercetin 3-O-robinobioside, and quercetin 3-O-arabinoglucoside) and quercetin monoglycosides (quercetin 3-O-galactoside, quercetin 3-O-glucoside, quercetin 3-O-arabinoside, and quercetin 3-O-xyloside) [48]. One of the key issues is the limited structural characterization of flavonoid glycosides. While some studies report profiles of quercetin and kaempferol derivatives, their complete glycosylation patterns and interactions with other plant bioactive compounds remain unclear [31,33]. Glycosylation strongly influences bioavailability and bioactivity; therefore, comprehensive characterization requires fully elucidated structures [49].

The branches of *A. alnifolia* contained the same groups of phenolic compounds as the leaves, but with higher amounts of flavan-3-ols and lower levels of flavonol glycosides and caffeoylquinic acids. This highlights the variation in phenolic compound levels within the same plant. The total amount of phenolic compounds in the branches was 500 mg/100 g of dry weight, compared to 1500 mg/100 g of dry weight in the leaves [50].

*A. alnifolia* was found to be rich in proanthocyanidins, which accounted for 3% of dry fruit biomass and 10–14% of dry biomass and leaves [2]. Leaves and stems contained more proanthocyanins than fruit (124.7 vs. 6.911 mg/g of dry weight). The proanthocyanidin concentration reached 1.363 mg/100 mL of berry juice. *A. alnifolia* has shown to have a high content of proanthocyanidins in the whole fruit (369.37 mg/100 g of fruit), comparable to that of strawberry, blueberry, chokeberry and sea buckthorn (446.72 mg/100 g, 258.60 mg/100 g, 285.91 mg/100 g, and 275.55 mg/100 g, respectively) [51]. Studies have found that the concentration of proanthocyanidins in fruit ranges from 32 to 27 mg/g of dry weight (depending on the cultivar), and it is lower in fruit than in leaves [28]. *A. alnifolia* fruit extracts contain proanthocyanidins ranging from dimers to heptamers and higher polymers. The proanthocyanidins identified were predominantly of the procyanidin type, consisting of epicatechin units linked by β-type bonds [15,39,52,53]. Unextractable proanthocyanidins, which remain in the residues after aqueous and organic extractions, were also found in *A. alnifolia*. Total unextractable procyanidins were determined to be 101 mg/100 g of fresh mass, accounting for over one-third of the total proanthocyanidins [54]. Therefore, research on the potential bioactivities of *Amelanchier* proanthocyanidins is still to be implemented. The polyphenol content of various *Amelanchier* species is shown in the table below (Table 1).

#### 2.2.3. Vitamin and Mineral Content of *Amelanchier* Species

The fruits of *A. alnifolia* were found to be rich in macroelements such as potassium, calcium, phosphorus, magnesium, and sulfur, while other macroelements were present only in trace amounts (below 10 mg/kg). The highest concentrations were observed for potassium (175–2034 mg/kg), calcium (477–676 mg/kg), phosphorus (392–484 mg/kg), and magnesium (288–371 mg/kg), highlighting the fruit’s potential as a good source of nutrients. Heavy metals such as lead, cadmium, and chromium were not detected in the samples, while only nickel and molybdenum were identified, though their levels remained within safe limits [58].

The mineral content of *A. alnifolia* fruits varied among the tested cultivars, showing genotype-specific differences in nitrogen, potassium, magnesium, phosphorus, calcium, and sodium [41]. Additionally, *A. alnifolia* was found to contain valuable vitamins. When compared to blueberries, *A. alnifolia* had higher levels of certain vitamins, including riboflavin (3.54 vs. 0.041 mg/100 g of fruit), vitamin A (10.91 vs. 3.0 mcg retinol activity equivalents/100 g of fruit), vitamin E (1.12 vs. 0.57 mg/100 g of fruit), and pantothenic acid (0.31 vs. 0.124 mg/100 g of fruit). These findings suggest that *Amelanchier* fruit could serve as a valuable vitamin source, comparable to commonly consumed fruits like blueberries [58].

#### 2.2.4. Sugar Content of *Amelanchier* Species

Mazza et al. reported the sugar content of *A. alnifolia* [35]. Compared to blueberries, *A. alnifolia* was found to have a higher total sugar content (15–20% vs. 14–17% of total weight). At maturity, glucose, fructose, and sorbitol account for 99% of the total sugar content in the fruit. Additionally, *A. alnifolia* contains higher levels of sucrose, glucose, and fructose than *V. corymbosum* (0.17, 5.23, and 5.94 mg/kg for *A. alnifolia* vs. 0.11, 4.88, and 4.97 mg/kg for *V. corymbosum*, respectively). These higher sugar levels contribute to the greater caloric value of *A. alnifolia* fruit (84.85 kcal/100 g vs. 57 kcal/100 g for blueberries), making it a potential ingredient for food products and nutritional applications. Sugar content varies between *A. alnifolia* cultivars, though the results remain similar. In the eight tested cultivars, glucose ranged from 4.03 to 4.70 g/100 g of fruit, while fructose ranged from 4.76 to 5.36 g/100 g of fruit. Understanding the nutraceutical and nutrient composition of *A. alnifolia* is essential for evaluating its benefits in the human diet [9,35,41].

#### 2.2.5. Terpenoid Content of *Amelanchier* Species

The triterpenoid content in *Amelanchier* fruit extracts varied across the studied genotypes. The total content ranged from 72.21 to 91.31 mg/kg of dry fruit mass, with an average of 79.0 mg/kg. The predominant compound was oleanolic acid, accounting for 84% of the total triterpenoid content, followed by betulinic acid (12%) and ursolic acid (5%). *A. alnifolia* fruit extracts contained ursolic acid, oleanolic acid, and betulinic acid in the ranges of 2.60–6.19, 56.02–87.83, and 6.67–11.62 mg/kg of dry mass, respectively. Various factors, including climate, environmental conditions, and fruit maturity at harvest influenced the total triterpenoid content. Donno et al. conducted a phytochemical analysis of *A. canadensis* fruits and identified a diverse profile of monoterpenes. Among the detected compounds were limonene, phellandrene, sabinene, γ-terpinene, and terpinolene, which are known for their potential antioxidant, antimicrobial, and anti-inflammatory properties [7]. Due to these aromatic compounds, *Amelanchier* fruits are used in cider, wine, beer, and tea production [4,15].

#### 2.2.6. Carotenoid Content of *Amelanchier* Species

In the *Amelanchier* species, the primary carotenoids identified include β-carotene and lutein, with minor amounts of zeaxanthin [38]. The concentration of carotenoids is genotype specific, with the greatest amounts found in the ‘clone type S’ cultivar of *A. alnifolia* (561.57 mg/kg of dry fruit mass), while the lowest carotenoid concentration was determined in the cultivar ‘Pembina’ (478.62 mg/kg of dry fruit mass). The primary compound was found to be β-carotene (68% of all carotenoid content) and lutein (32% of all carotenoid content). Another study compared the carotenoid content of fruits of different maturity stages in six *A. alnifolia* cultivars. Levels of lutein, zeaxanthin and β-carotene were found to be greater in green, unripe fruit than in the more mature fruit. The predominant carotenoid in all samples was determined to be lutein, with the greatest amounts being in mature fruit (ranging from 300 to 1000 mcg/100 g of fresh fruit). Zeaxanthin ranged from about 60 to 120 mcg/100 g of fresh fruit. These values are higher than the lutein levels reported for strawberries, lingonberries, cranberries, red currants and raspberries (31, 22, 28, 47, 76 mcg/100 g of fresh product, respectively). Although lutein concentration of *A. alnifolia* fruit extract was lower than some vegetables that are considered a great source of lutein (broccoli—1800 mcg/100 g; peas—1700 mcg/100 g; spinach—4400 mcg/100 g), it is nevertheless a good source of carotenoids and the further studies of its constituents and their biological effects should be considered [38,42,43].

#### 2.2.7. Cyanogenic Glycoside Content of *Amelanchier* Species

*A. alnifolia* seed extracts were reported to contain cyanogenic glycosides—naturally occurring compounds that can release toxic cyanide when metabolized. The toxicity of *A. alnifolia* plant material was explained in the toxicity section. The major cyanogenic glycosides identified were amygdalin and prunasin, with amygdalin consistently present at higher levels than prunasin. All detected levels were within non-toxic ranges. It was noted that the variation in results occurred due to seed size, as not all cultivar seeds were similarly sized. ‘Smoky’ and ‘Thiessen’ cultivars were determined to have the greatest levels of both glycosides. The hydrogen cyanide (HCN) potential was also considered; for ‘Smoky’ cultivar seeds, it was determined to be the highest (9.40 mg/kg of fresh weight), followed by the ‘Thiessen’ cultivar (8.15 mg/kg of fresh weight). The lowest HCN potential was determined in the ‘Honeywood’ cultivar, ranging from 0.39 to 4.24 mg/kg of fresh weight. In mature fruit extract, the content of amygdalin ranged from 43.0 to 129.0 mg/kg of fresh fruit, while the content of prunasin was 5–19 mg/kg of fresh fruit. These values are considerably lower than those of other fruits. For example, the prunasin content of passion fruit is 285 mg/kg, and the amygdalin level is 31 mg/kg. Bitter apricot kernels consist of 5.5 g/100 g of amygdalin content. It is worth mentioning that the cultivars that contained higher levels of amygdalin and prunasin were more aromatic and flavourful than other cultivars. This is because the major aroma component of these fruits is benzaldehyde. Amygdalin and prunasin possibly break down into benzaldehyde, hydrocyanic acid and glucose, although this requires further studies [43,59,60,61].

The minimum ingestion amounts to inflict acute toxicity on humans would theoretically be extremely large. A 100 kg person would need to ingest around 12 kg of fruit, and a 25 kg child would need around 3 kg of *Amelanchier* fruit of the varieties higher in cyanogen content. With proper care, moderation of consumption and awareness, these fruits can be safely ingested. Nevertheless, the data on the toxicity of the whole *Amelanchier* species are extremely limited, and even though acute toxicity is not likely, scientific uncertainties remain [43].

Environmental and developmental factors known from other cyanogenic glycoside containing plants, such as seasonal, phenological changes, plant stress, and soil conditions, could influence their amounts in plant materials of *Amelanchier*, but to our knowledge have not been systematically studied. Processing methods that reduce HCN potential in other *Rosaceae,* including thermal treatment and fermentation, remain untested for *Amelanchier* [62]. Importantly, no toxicological studies in humans, nor acute or chronic safety assessments of standardized *Amelanchier* extracts have been reported. Future research should therefore prioritize research on cyanogenic glycosides content across plant parts and phenological stages, and the evaluation of processing impacts to estimate bioavailable HCN release. Such data will be essential to ensure safe and sustainable use of *Amelanchier* in food and phytotherapy.

#### 2.2.8. Organic Acid Content of *Amelanchier* Species

Organic acids account for 22.63% of the total fruit content, with a total concentration of 350 mg/100 g of fresh fruit weight. Six organic acids were identified in *A. canadensis* extracts: citric, malic, oxalic, quinic, succinic, and tartaric acids. Among them, oxalic acid reached 18.65 mg/100 g, succinic acid 36.41 mg/100 g, and tartaric acid 295.57 mg/100 g of fresh fruit weight. Organic acids play a crucial role in fruit quality, stability, color, and flavor. Their presence and concentration are also used to assess fruit maturity, detect spoilage or adulteration in fruit juices, and serve as food acidifiers. Additionally, organic acids are valued for their antioxidative properties [7].

In *A. melanocarpa* and *A. ovalis*, oxalic, quinic, malic, shikimic, and citric acids were found in similar quantities. Malic acid was present in higher amounts in both species (4.8 and 3.5 g/100 g of dry fruit weight for *A. melanocarpa* and *A. ovalis*, respectively). Succinic acid was not detected in *A. ovalis*. However, oxalic acid was the only organic acid in *A. ovalis* that exceeded the concentration in *A. melanocarpa* (0.37 vs. 0.31 g/100 g of dry fruit weight) [36].

Other studies have shown that the predominant organic acid in *A. alnifolia* is succinate. However, succinate levels declined along with progressing fruit maturity, and malate was the predominant organic acid in mature fruit. Minor organic acids were also identified in the fruit, including quinate, galacturonate, citrate, pyruvate, *cis*-aconitate, fumarate and oxalate. Quinate concentration lowered when fruit reached later maturity stages (from 1.8 to 0.6 mg/g of fresh weight), along with galacturonate. Citrate declined about 2-fold (from 400 mcg to 200 mcg/g of fresh weight). Pyruvate was detectable in all fruit growth stages, although it was at its peak (225 mcg/g of fresh weight) during the fifth maturity stage (out of nine maturity stages). These results show that the concentration of organic acids changes during the fruit ripening process, and their effect on the biological activity of the fruit should be considered, as some organic acids are left with only trace amounts remaining [37].

#### 2.2.9. Fatty Acid Content of *Amelanchier* Species

Bakowska-Barczak & Kolodziejczyk analyzed the fatty acid content of *A. alnifolia* seed oil, identifying eleven fatty acids. Linoleic acid (18:2) was the most abundant, accounting for 55% of the total content, followed by oleic acid (18:1) at 32% and palmitic acid (16:0) at 6.7%. Among 17 tested cultivars, ‘Lee 3’ seed oil had the highest linoleic acid content (60.1%), while ‘Regent’ and ‘Success’ had the lowest (47.9% and 47.3%, respectively). Due to their lower linoleic acid content, Success’ and ‘Regent’ seed oils exhibited notably higher oleic acid levels compared to other cultivars. Conversely, ‘Lee 3’ seed oil, rich in linoleic acid, had the lowest oleic acid content (26%) [15]. All seed oils contained minimal amounts of R-linolenic acid, an n-3 essential fatty acid. The fatty acid profile of cultivated *A. alnifolia* seed oil closely resembled that of its wild counterparts. The saturated fatty acid levels were lower than those in pumpkin seed oil but higher than in hawthorn and blueberry seed oils [63]. The total unsaturated fatty acid content in the seed oils was high, ranging from 87.7% in ‘Success’ to 89.8% in ‘Parkhill’. However, significant differences were observed in mono- and polyunsaturated fatty acid content across cultivars. ‘Success’ and ‘Regent’ seed oils had the lowest levels of polyunsaturated fatty acids (48.2% and 48.9%, respectively), while ‘Lee 3’ and ‘Quaker’ had the highest (61.4% and 60.0%, respectively). These variations were linked to differences in linoleic acid, an n-6 essential fatty acid, which must be obtained through the diet as it cannot be synthesized by the human body. Since *A. alnifolia* seeds are by-products of fruit processing, exploring food applications for their oils could add value to fruit production [15,64].

### 2.3. Biological Activities and Traditional Uses of Amelanchier Species

**Figure 1:**
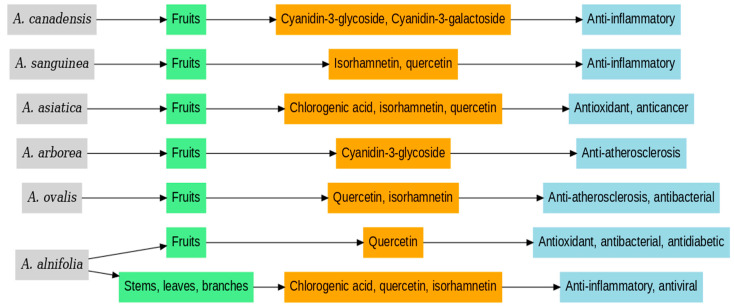
Bioactive compounds and biological activities in the main *Amelanchier* species.

*Amelanchier* plants thrive in various environments, and their adaptability makes them a popular choice for landscaping. Several species are planted for their fruit, which can be harvested and processed into jams, juices and other foods. The species are used not only for their ecological benefits but also for their biological activities (Figure 1).

The fruit, seeds, leaves and bark were found to contain antioxidants and phenolic acids known for their anti-inflammatory, anticancer, antibacterial and anti-diabetic properties. *Amelanchier* raw materials present considerable potential as a functional food or nutraceutical ingredient, particularly due to its dual antioxidant system (hydrophilic polyphenols together with lipophilic carotenoids and triterpenoids) [38]. Numerous studies have aimed to determine the biological activities of the extracts of fruits, leaves, and bark (Table 2) [2,4,15,28,36,63,65]. In addition, *Amelanchier* fruits are a good source of vitamins, particularly vitamins C and A, which play important roles in our immune function [35].

However, it is important to note that even though such activities and uses of the plants have been reported and studied, scientific research into the pharmacological properties of *Amelanchier* is still in its early stages, and more studies are needed to understand the full therapeutic potential and potential medicinal use of these plants. The fruits of *Amelanchier* were grown in orchards and traditionally used as a food source by Native Americans [32]. The fruits were used as juice for treating stomach and intestine ailments, eye drops were prepared from the fruit, the boiled bark was used as a disinfectant, and the root infusion was used to prevent miscarriage after injury. Tea was prepared from the twigs and administered to women after giving birth, and a tonic from the bark was used to discharge the placenta [78]. Tea from *Amelanchier* twigs and leaves was brewed to promote health and manage diabetes. American Indians continue to use plant sources like this to treat ailments like inflammation, diabetes and cancer [63]. Decoctions of the aerial parts of *A. alnifolia* were used to treat respiratory diseases, diarrhea, influenza and smallpox [75]. *Amelanchier*, dogwood and prickly rose roots are mixed to make medicine for venereal diseases, urinary tract infections and kidney problems. Native Americans often mix the dried fruit with animal fat and dried meat to make a high-energy food called pemmican [79]. The people of Central Mexico use the most widely distributed species—*M. denticulata* (=*A. denticulata*)—as fuel for fires, and branches are used as handles of small tools. The branches treated with lime are used in construction, offering support for roofs. The green leaves are used as feed for cattle. It was noticed that during the spring, this plant is preferred by bees, marking its importance in the production of honey. Fruits are used by the community as food; some communities harvest the fruit in order to preserve it in the form of jams [80].

#### 2.3.1. Antioxidant Properties

*Amelanchier* fruits are a rich source of antioxidants, primarily due to phenolic bioactive compounds including anthocyanins, phenolic acids, and flavonols [15,28,38]. Antioxidant capacity is largely linked to free radical scavenging activity, which correlates with the concentration of phenolic acids and anthocyanins [11]. In addition to these, other constituents such as triterpenoids (66.55–91.93 mg/kg of dry fruit mass) [38] and polysaccharides [81] contribute significantly to antioxidative potential. Purification of polysaccharide fractions enhances their ability to scavenge DPPH, hydroxyl, and ABTS+ radicals, with hydroxyl radical inhibition potentially involving glyoxylate–ferrous ion coupling [81].

Antioxidant potential differs considerably between *Amelanchier* species and plant parts. In comparative studies of *A. canadensis*, *A. humilis*, and *A. alnifolia* exhibited the highest total polyphenol content (187.49 Ru/100 g) and ferric-reducing antioxidant power (FRAP, 1610.49 AA/100 g) [31]. Within fruits, peels contained nearly 17-fold more flavonoids than pulp, though their ferric-reducing power was only about twice as high; the peel of *A. alnifolia* showed the highest antioxidant activity (14.5 mmol TE/100 g), with antioxidant levels in the peel averaging 5–16× higher than in flesh or seeds [31]. Variation is also observed between plant organs: *A. ovalis* leaves, twigs, young fruits, and flowers all show high radical scavenging capacity (92–93%), with total phenolic content ranging from 9.468 mg GAE/L in flowers to 32–37 mg GAE/L in other organs [82,83].

Cultivar differences are also notable. *A. alnifolia* ‘Nelson’ had the highest phenolic acid content (798.83 mg GAE/100 g), followed by ‘Honeywood’ (744.64 mg GAE/100 g), with corresponding antioxidant activities of 5.0 mmol TE/100 g and slightly lower values, respectively [15,28,38]. Phenolic content and antioxidant capacity are influenced by genotype, environmental conditions, and seasonal factors, with warmer, sunnier conditions promoting higher levels [84]. Post-harvest processing significantly impacts bioactivity: pasteurization of *A. canadensis* reduced anthocyanin content by half (from 53.2 mg/100 g) and nearly doubled the EC50 value in DPPH assays, while freezing caused only a slight decrease; optimized vacuum–microwave drying preserved ~70% of cyanidin glycosides [85,86].

*Amelanchier* extracts show promising applications in food products. In meat sausages, 3–5 mL/kg of *A. alnifolia* fruit extract reduced malondialdehyde (MDA) levels to values comparable with vitamin C supplementation (0.129–0.131 mg/kg vs. 0.128 mg/kg for vitamin C, compared with 0.165 mg/kg in controls) [87]. Wheat beers with *A. alnifolia* pulp had higher antioxidant capacity than control beers (2.94 vs. 2.27 mM TE/L in DPPH assay) [88]. Analyses show that *A. canadensis* has a FRAP value (25.07 ± 0.48 mmol Fe^2+^·kg^−1^) exceeding that of apple, raspberry, orange, and mulberry [7,78]. Its fruits contain substantial amounts of catechins (343.46 mg/100 g), anthocyanins (220.66 mg/100 g), and tannins (209.29 mg/100 g) [7,78]. *A. ovalis* inhibits lipid peroxidation at much lower concentrations than *A. melanocarpa* (0.35 ± 0.02 vs. 3.86 ± 0.12 mg/mL), suggesting cardiovascular benefits [36], and its extracts can extend yeast lifespan by >50% under oxidative stress [14]. Extracts of *A. alnifolia* fruits and leaves protect erythrocytes from oxidative damage [8], while *A. arborea* leaf extracts reduce neurotoxic effects in midbrain cultures, potentially via Nrf2 activation [89].

Extraction methods significantly affect yield and antioxidant activity. For *A. parviflora*, methanol extracts had the highest phenolic content (125.28 mg GAE/g) and antioxidant capacity (CUPRAC: 506.18 mg TE/g), compared to ethyl acetate extracts (60.18 mg GAE/g; 233.14 mg TE/g) [90]. *A. × lamarckii* also shows strong antioxidant potential (CUPRAC: 323.99 μmol TE/g) [12], and *A. asiatica* fruit extracts display significant DPPH activity [91].

Overall, available studies consistently demonstrate that *Amelanchier* extracts and isolated compounds possess notable antioxidant capacity, primarily attributed to their high hydroxycinnamic acid, flavonoid and anthocyanin content. However, most research was performed using in vitro chemical assays, with limited data from cellular or in vivo models, and no clinical studies to date. Methodological differences in extraction, quantification, and lack of phenological data challenge the direct comparison between studies.

#### 2.3.2. Anti-Inflammatory and Anticancer Properties

Anthocyanins, found in the species *Amelanchier*, are valued for their potential health benefits as antioxidants and anti-inflammatory agents. Three species of *Amelanchier* (*A. alnifolia, A. arborea* and *A. canadensis*) were investigated for their anti-inflammatory properties on cyclooxygenase (COX) enzymes. *A. arborea* contained the greatest amounts of anthocyanins (390 mg/100 g of fruit), compared to *A. canadensis* and *A. alnifolia*, 165 and 155 mg/100 g of fruit, respectively. The total anthocyanin content is comparable to that of other known and commonly used antioxidant fruits, like blueberries (total anthocyanin content 25–495 mg/100 g of fruit) [92]. COX-1 and COX-2 bind oxygen to the arachidonic acid, forming prostaglandin H2, which is further converted to various other prostaglandins that promote inflammation. COX-1 enzyme expression is common in most tissues, but cytokines and tumor growth factor mediate COX-2 induction. The studied species showed good COX enzyme inhibition, similar to that of anti-inflammatory drugs (aspirin, rofecoxib), and pure anthocyanins. The anthocyanin mixtures from the three species showed positive COX-1 and COX-2 inhibition results (60% inhibition of COX-1.72% inhibition of COX-2). The research shows the potential of using anthocyanin-containing *Amelanchier* species for their antioxidative properties in managing the mechanism of inflammation [4].

*A. asiatica* fruits were examined for their anti-inflammatory effects in a cell viability model on RAW 264.7 cells induced by lipopolysaccharide. Results showed 96% cell viability at 1.000 mcg/mL concentration. The result showed inhibition of NO production by inhibition of iNOS and COX-2 protein expression [22].

Proanthocyanidins, anthocyanins, quercetin derivatives, chlorogenic acid, benzoic acid, catechin (found in the stems), and epicatechin (specific to the leaves), which can be found in *A. alnifolia* were proven to be responsible for antioxidant, anti-inflammatory, antidiabetic properties, also they are responsible for lowering the risk of cardiovascular diseases [2,66,67]. *A. alnifolia* contains these substances in varying amounts (i.e., phenolic acids—1.43 mg/g, catechins 4.94 mg/g proanthocyanidins 98.54 mg/g) [2].

Nitric oxide (NO) is a free radical produced in mammalian cells and tissues, and in excess, it can contribute to tissue injury, oxidative stress and cancer [65]. Flavonoids, found in blueberries and the fruit of *A. alnifolia* inhibited NO production in bacterial lipopolysaccharide/interferon-γ activated RAW264.7 macrophages [28,65]. The increased production of tumor necrosis factor α in lipopolysaccharide/interferon-γ activated macrophages could have chemopreventive activity because the TNF-α from the active macrophages leads to cytostatic and cytotoxic activities on malignant cells. This can contribute to the antitumor properties of anthocyanin extracts of *A. alnifolia* [68].

Cytoprotective assays have been carried out with the human hepatocellular liver carcinoma cell line hepG. These cells were pre-treated with 100 µg/mL/ of the extract of *A. alnifolia* fruit extracts and then tested to determine their viability against tert-butylhydroperoxide. Significant cytoprotection was found in five cultivars of *A. alnifolia*—‘Velva Martin’, ‘Wild type KH4’, ‘Wild type NT3’, ‘WC 3A’ and ‘WC 2B’ (48%, 46%, 42%, 55% and 55%, respectively). The total phenolic content of these cultivars was also among the greatest ones (7.813, 19.311, 10.984, 5.530, and 6.234 mg/g, respectively), suggesting that the phenolic content of the plant material is responsible for its cytoprotective effects on cells [69].

*A. sanguinea* fruits, along with others (cranberry, strawberry, black currant, raspberry, blueberry, gooseberry) were tested for their anticancer effects on breast cancer MDA-MB-231 cells and prostate cancer PC-3 cells. *A. sanguinea* fruit juice was a weak inhibitor of cancer cell proliferation (0–30% inhibition at 60 mcl/mL) in both cases. Still, it was one of the fruits tested that had a high antioxidant capacity (14.6 µmol TE/mL). Although showing weak inhibition, it could still prove useful for the prevention of tumor development [70].

Extracts from mainly *Amelanchier* fruits have demonstrated the ability to inhibit pro-inflammatory mediators and pathways in cell-based models. Still, the compound–activity relationships remain unclear, and the responsible compounds have not been clearly elucidated. Furthermore, the other parts of the plant remain uninvestigated.

#### 2.3.3. Antidiabetic Properties

Ethnopharmacological data confirm that *A. alnifolia* was used by the Blackfeet Indian tribe for the treatment of diabetes [93]. Zhang et al. have studied the potential use of *A. alnifolia* leaf extract on obese and hyperglycemic mice prior to carbohydrate loading. The leaf extracts demonstrated significant inhibition of α-glucosidase activity; the absorption of carbohydrates was delayed, and thus, the blood glucose concentration was significantly lowered. The extracts suppressed glucose absorption by inhibiting the sucrose, maltose and isomaltose activities of the small intestinal α-glucosidases [63]. Other studies have reviewed the use of *A. alnifolia* fruit powder for use against the symptoms of metabolic syndrome. The mechanism for the improved metabolic response while administering the fruit powder could be improved glucose regulation. In the liver, protein kinase B regulates glucose metabolism by assisting the conversion of glucose to glucose 6-phosphate by stimulating the expression of hexokinase. Protein kinase B increases the translocation of the protein glucose transporter 1 to the plasma membrane and stimulates glycogen synthesis. The supplementation with *A. alnifolia* normalized the expression of hexokinase 1 (the enzyme that phosphorylates glucose for utilization in glycolysis and glycogenesis) and increased the expression of glucose 6-phosphatase, which in turn increased glycolysis and gluconeogenesis. This supplementation resulted in decreased cardiac inflammation, normalized body weight, improved glucose tolerance, and decreased systolic blood pressure and total cholesterol [71,72]. Kraft et al. examined the fruit of four wild species, including *A. alnifolia*, to assess the health benefits of their phytochemicals. They conducted several biological tests to evaluate the potential impact of the fruit on various microvascular complications of diabetes, such as hyperglycemia, the expression of pro-inflammatory genes, and symptoms of metabolic syndrome. Nonpolar compounds found in berries, such as carotenoids, were effective inhibitors of aldose reductase (the test showed inhibition of 82%), an enzyme linked to the development of diabetic microvascular complications. In contrast, the polar compounds, primarily phenolic acids, anthocyanins, and proanthocyanidins, acted as hypoglycemic agents and were strong inhibitors of the gene expression for IL-1β (inhibited by 36%) [55,72].

Overall, the available evidence suggests that *A. alnifolia* may exert antidiabetic effects through multiple mechanisms, including inhibition of intestinal α-glucosidases, modulation of hepatic glucose metabolism, and suppression of pro-inflammatory pathways. However, most of the current data are using crude extracts or powders without standardized phytochemical profiles. Future studies are needed to clarify the relative contributions of phenolic, carotenoid, and other metabolite classes to glycemic control and the prevention of diabetic complications.

#### 2.3.4. Antibacterial Properties

Research towards the antimicrobial activity of underutilized plant extracts is important for developing sustainable, plant-based alternatives to synthetic antibiotics, potentially offering broad-spectrum activity against various pathogens while contributing to environmentally friendly and effective therapeutic solutions [94]. Sagandyk et al. have studied the antibacterial properties of *A. melanocarpa* and *A. ovalis*. The antimicrobial mechanism of action involves several pathways—damage to the bacterial cell wall membranes, which leads to increased cell wall permeability, nucleic acid and protein synthesis inhibition, and disruption of mitochondrial functions. Although *A. melanocarpa* showed superior antimicrobial activity, requiring lower concentrations of its extract, *A. ovalis* was also effective against both Gram-positive and Gram-negative strains of bacteria. The antimicrobial effects against the species of bacteria are attributed to the anthocyanins and non-anthocyanin phenolic compounds present in the composition of the plant extracts [36]. Jantová et al. studied *A. ovalis* and various other species of plants to test their antimicrobial properties. *A. ovalis* has shown the highest antibacterial effect on Gram-positive bacteria (inhibition of growth of *Staphylococcus aureus*—38.2%; *Enterococcus faecalis—*34.0%) [73]. Lachowicz et al. have found that the fruit extracts from *A. alnifolia* species had a genotype—dependent significant impact on inhibiting the growth of *Enterococcus hirae*. The strong inhibition of the microorganism growth was linked to the high content of polyphenolic compounds, nucleotides, and free amino acids in the fruit samples [31]. Tian et al. have studied the antimicrobial effects of *A. alnifolia* fruits, leaves and branches, and determined antimicrobial activity against *E. coli* (57%, 75%, 68% for the fruit, leaves and branches respectably), *S. aureus* (31%, 100%, 100% for the fruit, leaves and branches respectably), *L. monocytogenes* (74%, 100%, 100% for the fruit, leaves and branches respectably), *B. cereus* (6%, 89%, 84% for the fruit, leaves and branches, respectively) [50].

Mostly fruit samples have been predominantly tested to date, and future research should extend antimicrobial assessments to other plant parts, such as leaves, bark, and seeds, which may possess distinct and potentially stronger bioactive profiles. Invasive *Amelanchier* species could also be of interest, as their utilization for bioactive compound extraction may provide both therapeutic value and ecological management benefits.

#### 2.3.5. Antiviral Properties

The antiviral properties of *Amelanchier* species have not been thoroughly studied through molecular screening of the phytochemicals from *A. alnifolia* against hepatitis C virus (HCV) NS3/4A protease and helicase. Molecular docking simulations of flavonoids with HCV target proteins show the good binding ability of *A. alnifolia* derived flavonoids quercetin 3-galactoside and 3-glucoside with protease and helicase of HCV, thus providing insights to consider these flavonoids as potential inhibitors of HCV target proteins [74]. *A. alnifolia* twig methanolic extract was determined to completely inhibit the cytopathic effects of an enteric coronavirus [75,76].

#### 2.3.6. Other Properties

The fruit extracts of *A. × lamarckii* have been found effective at inhibiting key enzymes like tyrosinase and acetylcholinesterase. These enzymes are responsible for skin pigmentation and neurodegenerative effects. Literature data suggests that polyphenols like quercetin, isorhamnetin and gallic acid are responsible for the inhibition activity of tyrosinase [12]. The fruit extract obtained from *A. parviflora* was proven to have anti-tyrosinase (145.54 mg KAE/g extract) and anti-acetylcholinesterase (3.63 mg GalE/g extract) activity [12,77]. Adding *A. alnifolia* fruit powder to the wheat flour of cookie dough seemed to improve some technological parameters (decreasing gluten content). A positive nutritional value was influenced by increased ash content when fruit powder was added. The reduction in water activity value has a positive influence on shelf life. Adding fruits also improved the appearance and taste of the cookies examined [95].

#### 2.3.7. Toxicity of *Amelanchier* Species

Despite the positive effects described above, it is recognized that *A. alnifolia* can produce toxic levels of hydrogen cyanide (HCN). Prunasin has been found in the twigs of *A. alnifolia*. Its levels are at their highest during the growth of new twigs (3.37% vs. 2.23% of later grown twigs and 2.32% of old wood), although all types of twigs were considered as dangerous for cattle, as a 1.4% concentration of prunasin is required for acute poisoning [60]. In *Rosaceae*, cyanogenic glycosides are common defense metabolites also found in *Prunus* or *Sorbus*, where their toxicological profiles are better characterized [96,97]. Two cultivars of *A. alnifolia* were also studied for their prunasin contents (*A. alnifolia* var. *alnifolia* and *A. alnifolia* var. *cusickii* (*=A. cusckii*). The var. *alnifolia* has a much lower HCN potential than var. *cusickii*. The average prunasin contents of *A. cusickii* leaves were threefold higher than those of var. *alnifolia* (6.45% vs. 1.88% of prunasin) [61]. This indicates that the toxicity of the species can vary significantly between different cultivars of the plant. The species has been proven to be toxic to livestock in vivo. Hydrogen cyanide is generated enzymatically from cyanogenic glycosides of *A. alnifolia* when plant tissue is chewed. In vivo experiments were used to demonstrate this toxicity. Chopped *A. alnifolia* twigs were fed as 75% of the diet. This amount was enough to provide evidence of cyanide poisoning, as the cattle exhibited restlessness, shivering and shortness of breath, increased heart rate and weight loss. Venous blood and rumen fluid were obtained to provide proof of hydrogen cyanide poisoning. It is approximated that toxicity does not occur if the cyanide level is less than 5 mg HCN/100 g. *A. alnifolia* fruit showed a level that did not exceed 2 mg HCN/100 g of fruit. In comparison, leaves had an HCN potential of 101.9 mg HCN/100 g, and fresh wood had a potential of 484.8 mg HCN/100 g. These results indicate that vegetative parts of *A. alnifolia* are potentially hazardous to ruminants, although the fruit can be considered safe [60]. However, to the best of our knowledge, no data on the toxicity of Amelanchier genus plants to humans were found, indicating the need for further studies.

## 3. Materials and Methods

The literature search was performed using Google Scholar, PubMed, Springer link, ScienceDirect, Scopus, and World Flora Online search engines, with a time limit from 1946 to 2025. Keywords “*Amelanchier*”, “*Amelanchier alnifolia*”, “*Amelanchier canadensis*”, “shadbush”, “saskatoon”, “serviceberry”, and “juneberry was combined with “phytochemistry”; “traditional medicine”, “biological activity”, “toxicology”, phenolic compounds”, “flavonoids”, “proanthocyanidins”, “triterpenoids”, “carotenoids”, “sugars”, “organic acids”, “vitamins”, etc. Finally, 101 original articles about *Amelanchier* from this period were included in this review.

## 4. Conclusions and Future Directions

The present review compiles current knowledge on the phytochemistry and bioactivity of *Amelanchier* species, highlighting their rich content of phenolic compounds, triterpenoids, carotenoids, vitamins, minerals, and unsaturated fatty acids, as well as their diverse biological effects. While these findings confirm the genus as a promising source of functional and therapeutic agents, they also reveal several research gaps that limit a comprehensive understanding of *Amelanchier* as a resource for functionally essential compounds. Firstly, available data is targeted toward a small number of agriculturally utilized species with particular attention on *A. alnifolia*. The phytochemical and biological activity potential of many taxa remain underexplored. Quantitative phytochemical studies often lack clear differentiation between fresh and dry material and an indication of phenological stage, which significantly impacts the phytochemical profiles. Agronomic, ecological, and post-harvest factors affecting metabolite content also should be considered. Bioactivity research is predominantly based on antioxidant measurements, with limited mechanistic insights or in vivo testing. Moreover, research has concentrated almost exclusively on fruits, leaving other potentially valuable organs such as leaves, seeds, and bark largely uninvestigated. Addressing these gaps will require multidisciplinary approaches linking taxonomy, chemistry, biology, safety, and valorization practices.

Future research should aim to address these gaps through multidisciplinary approaches. Expanding studies to include lesser-known species and cultivars, as well as multiple plant organs and developmental stages, would provide a more complete picture of *Amelanchier’s* phytochemical and functional potential. Phytochemical analyses should be systematically linked to cultivation conditions, harvest timing, storage, and extraction methods to identify factors influencing metabolite composition and stability. Biological studies should employ phytochemically characterized extracts or fractions in in vivo pharmacological models, combined with mechanistic investigations and pharmacokinetic profiling, to elucidate the key therapeutic *Amelanchier* compounds. Furthermore, agronomic and ecological research could assess invasive potential, and support the valorization of underutilized plant parts such as leaves, seeds, and bark. Addressing these gaps will require multidisciplinary approaches linking taxonomy, phytochemistry, pharmacology, safety assessment, and valorization strategies. Future research should:

- Expand studies to lesser-known *Amelanchier* species and cultivars, as well as different plant organs and developmental stages.
- Systematically connect phytochemical analyses with cultivation conditions, harvest timing, storage, and extraction methods to better understand metabolite variability and stability.
- Employ phytochemically characterized extracts or fractions in in vivo pharmacological models, complemented by mechanistic and pharmacokinetic studies, to identify bioactive constituents and clarify therapeutic potential.
- Explore agronomic and ecological aspects, including invasive potential and sustainable valorization of underutilized plant parts.
- Integrate ethnobotanical knowledge with modern analytical approaches to broaden the medicinal, nutritional, and ecological applications of *Amelanchier*.

Finally, the investigation of the *Amelanchier* species is scarce due to several limitations: limited geographic distribution, taxonomic complexity, lack of economic interest, underutilization in medicine and industry, and invasive status. By bridging traditional ethnobotanical knowledge with modern phytochemical research, *Amelanchier* species hold significant potential as multi-functional resources in medicinal, nutritional, and ecological applications.

## Figures and Tables

**Table 1:** Polyphenol content in different parts of *Amelanchier* species.

Compounds	Plant Part	Solvent Used for Extraction	Species	Detection Method	Country	References
(+)-Catechin (-)-Epicatechin 5-O-caffeoylquinic acid 3-O-caffeoylquinic acid Quercetin 3-O-xyloside Cyanidin 3-O-galactoside Cyanidin 3-O-arabinoside	Fruits, leaves	70% ethanol	*A. alnifolia*	UPLC-DAD-MS	Finland	[50]
5-O-caffeoylquinic acid 3-O-caffeoylquinic acid Cyanidin 3-O-galactoside Cyanidin 3-O-glucoside Quercetin 3-O-galactoside Quercetin 3-O-arabinoside	Fruits	acetone: methanol: water: formic acid (40:40:20:0.1 *v*/*v*/*v*/*v*)	*A. alnifolia*	HPLC, LC-MS	Canada	[46]
*cis*-5-Caffeoylquinic acid Caffeic acid hexoside *p*-Coumaric acid hexoside Ferulic acid hexoside 1 Ferulic acid hexoside 2 Chlorogenic acid Gallic acid Apigenin dirhamnoside Apigenin hydroxyhexoside Epicatechin Catechin Isorhamnetin-3-rutinoside Kaempferol-3-galactoside Kaempferol-3-glucoside Kaempferol-3-rutinoside Kaempferol pentoside 1 Kaempferol pentoside 2 Kaempferol dirhamnoside Kaempferol coumaroyl acetylrhamnoside Kaempferol rhamnoside Kaempferol rhamnosyl pentoside 1 Kaempferol rhamnosyl pentoside 2 Kaempferol rhamnosyl hexoside 1 Kaempferol rhamnosyl hexoside 2 Kaempferol rhamnosyl hexoside 3 Quercetin-3-galactoside Quercetin-3-glucoside Quercetin-3-rutinoside Quercetin-3-rhamnoside Quercetin-3-arabinopyranoside Quercetin dirhamnoside Quercetin glycoside 1 Quercetin glycoside 2 Quercetin rhamnosyl pentoside Quercetin coumaroyl acetylrhamnoside Cyanidin-3-galactoside Cyanidin-3-glucoside Cyanidin-3-xyloside Peonidin-3-glucoside	Fruits	methanol: water: formic acid (70:27:3, *v*/*v*/*v*)	*A. × lamarckii*	PDA-HPLC MS	Slovenia	[33]
Cyanidin-3,5-O-diglucoside Neochlorogenic acid Caffeoylglucose Chlorogenic acid Cryptochlorogenic acid Procyanidin B2 *p*-Coumaroylglucoside Cyanidin-3-O-galactoside Cyanidin-3-O-glucoside (-)-Epicatechin Cyanidin-3-O-arabinoside Cyanidin-3-O-xyloside Quercetin-3-O-vicianoside Quercetin-3-O-robinobioside Quercetin-3-O-galactoside Quercetin-3-O-glucoside Kaempferol-O-hexoside-O-pentoside Kaempferol-O-hexoside-O-rhamnoside Quercetin-3-O-arabinoside Dicaffeoylquinic acid Kaempferol-3-O-rhamnoside-7-O-glucoside Quercetin-3-O-rhamnoside Isorhamnoside-3-O-rutinoside Kaempferol-3-O-arabinoside Dicaffeoylquinic acid Kaempferol-3-O-rhamnoside	Fruits, leaves	water containing 200 ppm SO_2_	*A. alnifolia*	UPLC-DAD UPLC-ESI-MS	Poland	[8]
Cyanidin 3-galactoside Cyanidin 3-glucoside Cyanidin 3-arabinoside Cyanidin 3-xyloside	Fruits	100% ethanol	*A. alnifolia*	HPLC-DAD	Canada	[30]
Chlorogenic acid Caffeic acid Hydroxybenzoic acid Cyanidin 3-glucoside Petunidin 3-glucoside Cyanidin 3,5-diglucoside Cyanidin 3-xyloside Catechin Epicatechin	Fruits	80% ethanol	*A. alnifolia*	ESI-MS	USA	[55]
Chlorogenic acid Neochlorogenic acid Dicaffeoylquinic acid Cyanidin-3,5-diglucoside Cyanidin-3-galactoside Cyanidin-3-glucoside Cyanidin-3-arabinoside Cyanidin-3-xyloside Quercetin-3-vicianoside Quercetin-3-robinobioside Quercetin-3-galactoside Quercetin-3-glucoside Quercetin-3-pentoside Quercetin-3-pentoside	Fruits	80% ethanol with 0.1% formic acid	*A. alnifolia*	HPLC-ESI-MS/MS	Canada	[15]
Protocatechuic acid Neochlorogenic acid *p*-Hydroxybenzoic acid Chlorogenic acid Galic acid Cryptochlorogenic acid 4-Caffeoylquinic acid Caffeic acid glucoside Dicaffeic acid Kampferol-3-galactoside Quercetin-3-O-arabinoglucoside Quercetin-3-O-rutinoside Quercetin-3-O-galactoside Quercetin-3-O-glucoside Quercetin-3-O-rabinobioside Quercetin-3-O-arabinoside Quercetin-3-O-xyloside Quercetin-deoxyhexo-hexoside (+)-Catechin (−)-Epicatechin Cyanidin-3-O-galactoside Cyanidin-3-O-glucoside Cyanidin-3-O-arabinoside Cyanidin-3-O-xyloside	Fruits	methanol with 2.0% formic acid	*A. alnifolia*	UPLC-PDA-Q/TOF-MS	Poland	[38]
Cyanidin-3-glucoside Cyanidin-3-rutinoside Delphinidin-3-glucoside Delphinidin-3-rutinoside Delphinidin-3-galactoside Petunidin-3-galactoside Petunidin-3-glucoside Malvidin-3-glucoside Malvidin-3-galactoside Malvidin-3-arabinoside Peonidin-3-arabinoside Peonidin-3-galactoside	Fruits	methanol (1N HCL) (85:15) (*v*/*v*)	*A. alnifolia*	UPLC-ESI-MS/MS	Canada	[51]
Cyanidin-3-galactoside Cyanidin-3-glucoside Cyanidin-3-arabinoside Cyanidin-3-xyloside	Fruits	80% methanol	*A. alnifolia*	HPLC-ESI-MS/MS	Canada	[15]
Cyanidin-3-galactoside Cyanidin-3-glucoside Cyanidin-3-arabinoside Cyanidin-3-xyloside	Fruits	acetone: methanol: water (35:35:30) acidified with 1 mL of HCl at 36%	*A. alnifolia*	HPLC	Poland	[10]
Delphinidin-3-O-glucoside Cyanidin-galactoside Delphinidin-3-O-arabinoside Cyanidin-glucoside Cyanidin-arabinoside 5-Caffeoylquinic acid 4-Caffeoylquinic acid 3-Caffeoylquinic acid Quercetin-arabinoglucoside Quercitin-galactoside	Fruit Pomace	60%, 70%, 80%, 100%, (*v*/*v*) with 0.15 N HCl	*A. alnifolia*	HPLC	USA	[56]
3,4-Dihydroxy-5-methoxybenzoic acid Cyanidin-galactoside Chlorogenic acid Luteolin-rutinoside Cyanidine 4-Hydroxybenzoic acid-glucoside Feruloylquinic acid Quercetin-dirhamnoside Quercetin-rhamnoside Kaempferol-glucoside Dicaffeoylquinic acid Kaempferol-rhamnoside Luteoline Quercetin Kaempferol	Fruits	acidified methanol (0.3% with HCl)	*A.× lamarckii*	LC-ESI+-MS	Romania	[12]
Caffeic acid Chlorogenic acid Coumaric acid Ferulic acid Hyperoside Isoquercitrin Quercetin Quercitrin Rutin Ellagic acid Gallic acid Catechin Epicatechin Castalagin Vescalagin	Fruits	juice diluted in distilled water, by titration with 0.2 M NaOH	*A. canadensis*	HPLC-DAD	Italy	[7]
Neochlorogenic acid (+)-Catechin Chlorogenic acid (-)-Epicatechin Quercetin	Fruits, leaves, stems	1 mL of acidified acetone (1% formic acid in 70% acetone)	*A. alnifolia*	HPLC-DAD HPLC-ESI/MS	Finland	[2]
Neochlorogenic acid Chlorogenic acid 4-O-Caffeoylquinic acid Coumaric acid Coumaroylquinic acid 1,5-Dicaffeoylquinic acid Protocatechuic acid Rutin Isorhamnetin-3-rutinoside Kaempferol-3-O-rutinoside Isoquercitrin Hyperoside Quercetin-3-arabinoside-7-glucoside Kaempferol-3-sambubioside Quercetin-3-O-robinobioside Reynoutrin Astragalin Quercetin-3-O-malonylglucoside Quercetin-3-O-α-L-arabinopyranoside Isorhamnetin-3-O-glucoside Quercitrin Kaempferol-3-O-arabinoside Kaempferol-3-O-acetyl-glucoside Isorhamnetin pentoside Afzelin Quercetin-3-O-acetyl-rhamnoside Kaempferol-3-O-(6-acetyl-galactoside)-7-O-rhamnoside (−)-Epicatechin	Leaves	70% ethanol	*A. × spicata*	HPLC- MS HPLC- PDA	Lithuania	[57]

**Table 2:** Reported biological activities, bioactive constituents, and mechanisms of action of *Amelanchier* species.

Species	Material	Model	Concentration	Putative Compounds	Mechanism of Action	References
*A. alnifolia*	Fruits	In vitro antioxidant assays (DPPH, FRAP)	5.0 mmol TE/100 g fruit	Quercetin, chlorogenic acid, cyanidin-3-galactoside, cyanidin-3-glucoside	Free radical scavenging, ferric ion reduction	[15,28,38]
*A. ovalis*	Fruits	Lipid peroxidation inhibition assay	0.35 ± 0.02 mg/mL (IC50)	Quercetin, rutin, chlorogenic acid	Inhibits lipid peroxidation (anti-atherosclerosis)	[36]
*A. ovalis*	Fruits	Yeast oxidative stress model	Not stated	Quercetin, rutin, chlorogenic acid	Prevents H_2_O_2_-induced oxidative stress; increases yeast cell lifespan	[14]
*A. arborea*	Fruits	COX enzyme inhibition	390 mg anthocyanins/100 g	Cyanidin-3-galactoside, cyanidin-3-glucoside	Inhibits COX-1 & COX-2 (reduces prostaglandin-mediated inflammation)	[4]
*A. asiatica*	Fruits	RAW 264.7 macrophages	1 μg/mL	Cyanidin-3-glucoside, chlorogenic acid, quercetin	Inhibits NO production via suppression of iNOS & COX-2 expression	[22]
*A. alnifolia*	Fruits, stems, leaves	In vitro assays	Not stated	Catechin, epicatechin, chlorogenic acid, cyanidin glycosides	Antioxidant, anti-inflammatory, antidiabetic, cardioprotective	[2,66,67]
*A. alnifolia*	Fruits	RAW 264.7 macrophages	Not stated	Cyanidin glycosides, quercetin	Inhibits NO production; TNF-α-mediated cytotoxicity on cancer cells	[28,65,68]
*A. alnifolia*	Fruits	HepG2 cell cytoprotection	100 μg/μL	Quercetin, chlorogenic acid, cyanidin glycosides	Protection against tert-butylhydroperoxide-induced damage	[69]
*A. sanguinea*	Fruits	MDA-MB-231 & PC-3 cancer cell lines	60 μL/mL	Quercetin, chlorogenic acid, cyanidin glycosides	Weak inhibition of proliferation; high antioxidant activity	[70]
*A. alnifolia*	Leaves	In vivo (mice)	Not stated	Quercetin, rutin	α-glucosidase inhibition, delayed carbohydrate absorption	[63]
*A. alnifolia*	Fruits	In vivo (mice with metabolic syndrome)	Not stated	Quercetin, lutein, β-carotene	Improved glucose metabolism via PKB activation, reduced inflammation	[71,72]
*A. alnifolia*	Fruits	In vitro aldose reductase inhibition	82% inhibition	Quercetin, gallic acid, cyanidin glycosides	Prevention of diabetic microvascular complications	[55,72]
*A. ovalis*	Fruits	Antibacterial assays	Not stated	Cyanidin glycosides, quercetin, chlorogenic acid	Cell wall disruption, protein/nucleic acid synthesis inhibition	[36]
*A. ovalis*	Fruits	Antibacterial assays	Not stated	Quercetin, rutin, chlorogenic acid	Inhibition of Gram-positive bacterial growth (*S. aureus*, *E. faecalis*)	[73]
*A. alnifolia*	Fruits	Antibacterial assays	Not stated	Quercetin, rutin, amino acids (arginine, lysine)	Inhibition of *Enterococcus hirae* growth	[31]
*A. alnifolia*	Fruits, leaves, branches	Antibacterial assays	Not stated	Quercetin, rutin, chlorogenic acid	Inhibition of *E. coli*, *S. aureus*, *L. monocytogenes*, *B. cereus*	[50]
*A. alnifolia*	Twigs	Antiviral assay (enteric coronavirus)	Complete inhibition	Quercetin glycosides	Inhibition of viral protease and helicase activity	[74,75,76]
*A. × lamarckii*	Fruits	Enzyme inhibition assays	Not stated	Quercetin, isorhamnetin, gallic acid	Tyrosinase inhibition; acetylcholinesterase inhibition	[12]
*A. parviflora*	Fruits	Enzyme inhibition assays	145.54 mg KAE/g extract; 3.63 mg GalE/g extract	Quercetin, gallic acid, isorhamnetin	Anti-tyrosinase; anti-acetylcholinesterase	[12,77]
*A. alnifolia* var. *alnifolia,* var. *cusickii*	Twigs, leaves, wood,	In vivo (cattle)	Twigs: up to 3.37% prunasin; Leaves: 101.9 mg HCN/100 g; Wood: 484.8 mg HCN/100 g	Prunasin	Acute cyanide poisoning in cattle at ≥ 1.4% prunasin	[60,61]
